# Prognosis of coronary heart disease after percutaneous coronary intervention: a bibliometric analysis over the period 2004–2022

**DOI:** 10.1186/s40001-023-01220-5

**Published:** 2023-09-01

**Authors:** Shiyi Tao, Xianwen Tang, Lintong Yu, Lingling Li, Gaoyu Zhang, Lanxin Zhang, Li Huang, Jiayun Wu

**Affiliations:** 1 Graduate School, Beijing University of Chinese Medicine, Beijing, China; 2 Department of Cardiology, Beijing University of Chinese Medicine Shenzhen Hospital (Longgang), Shenzhen, Guangdong China; 3 The First Hospital of Hebei Medical University, Shijiazhuang, Hebei China; 4 Department of Oncology, Guang’anmen Hospital, China Academy of Chinese Medical Sciences, Beijing, China; 5 Department of Integrative Cardiology, China-Japan Friendship Hospital, Beijing, China

**Keywords:** Coronary heart disease, Percutaneous coronary intervention, Bibliometrics, CiteSpace, Visualization analysis

## Abstract

**Background:**

As the complexity and diversity of the percutaneous coronary intervention (PCI) are being explored and reported, burgeoning research has progressed in this field. However, there is no comprehensive analysis available on PCI-related studies published in the literature. This study aimed to analyze and visualize the changes of scientific output regarding prognosis of coronary heart disease (CHD) after PCI over the past 20 years and to reveal the knowledge domain and development trends in this field by using CiteSpace software.

**Methods:**

Relevant articles published over the period 2004–2022 were retrieved from the Web of Science Core Collection database. After manual selection, qualified documents were included and recorded with the information of their title, abstract, keyword, author, descriptor, citation, identifier, publishing year and publishing organization. We transferred the data to CiteSpace V5.8.R2 (Version 5.8.R2) to draw knowledge maps and to conduct co-occurrence analysis, cluster analysis, timeline analysis, burst term detection and citation analysis.

**Results:**

A total of 14,699 literature records were found relating prognosis of CHD after PCI in the past 20 years (2004–2022), including 14,212 original articles and reviews, and they were published in 153 different journals. Publication production has increased annually and a total of 1182 authors, 796 institutes and 147 countries have contributed to these publications. Moreover, the most representative author was Gregg W Stone from the CardioVascular Research Foundation (CVRF) with 368 publications, whose team mainly focused on exploring the efficacy and safety of revascularization and the characteristics of susceptible population. The global productivity ranking was led by the USA with 3326 published papers, followed by Italy (n = 1355), Japan (n = 1080), China (n = 1075) and Germany (n = 937). And the keywords of these publications were “percutaneous coronary intervention” (n = 2271), “outcome” (n = 1756), “mortality” (n = 1730) and “impact” (n = 1334). Other commonly-used words were “predictor” (n = 1324), “intervention” (n = 1310), “angioplasty” (n = 1299), “risk” (n = 1144), “acute myocardial infarction” (n = 1136) and “artery disease” (n = 1098). Cluster analysis showed that 15 high connected clusters were generated with a modularity Q of 0.831 and a weighted mean silhouette of 0.9388 by applying the log-likelihood ratio algorithm, and the top 5 clusters were #0 optical coherence tomography, #1 dual antiplatelet therapy, #2 bleeding, #3 clopidogrel and #4 thrombus aspiration. Furthermore, the frontiers in the field of prognosis of CHD after PCI mainly involved “decision making”, “reperfusion”, “angioplasty”, “balloon”, “unstable angina”, “dual antiplatelet therapy”, “cardiac surgical score”, “restenosis”, “reperfusion”, “thrombolytic therapy”, etc.

**Conclusions:**

To sum up, efficacy and safety of different types of stents, the risk factors of restenosis and thrombotic events after PCI, early risk assessment, and secondary prevention and complications of patients with CHD after PCI were research hotspots and frontier topics in the area by bibliometric analysis. The results could provide a comprehensive overview of the research hotspots and frontier topics relating prognosis of CHD after PCI, promoting a better understanding of the knowledge domain and development trends in this field during the past 20 years.

## Background

Coronary heart disease (CHD) is a type of ischemic heart disease characterized by atherosclerotic plaque accumulation in the coronary arteries [1]. As one of the leading causes of hospitalization and patient death [2], CHD has affected over 110 million individuals worldwide [3] and has been a heavy burden on health expenditure [4]. Percutaneous coronary intervention (PCI) technique has developed rapidly in past decades and now is a main treatment for patients with CHD. It has the advantages of restoring cardiac blood perfusion, ameliorating clinical symptoms, preventing disease progression and reducing short-term mortality as well [5]. However, adverse cardiovascular events (ACE) still occur in some CHD patients after PCI even though they have been administered with regular secondary prevention treatment after the surgery. Previous studies have shown that the incidence of cardiovascular end-point events such as post-procedure myocardial infarction, revascularization and all-cause death is about 5% to 15% [6–8]. It is reported that recurrent rate of chest pain is as high as 50% [9]. These adverse events pose a great threat to patient health and life, leading to an increase in the amount spent on healthcare due to growing re-hospitalization. Nowadays, countries around the world have paid more and more attention to the prognosis of diseases, especially CHD after PCI [10]. Thus, it is very important for researchers to identify the current knowledge domain and development trends in the area of prognosis of CHD after PCI.

CiteSpace is a software for literature visualization, which could be utilized to conduct bibliometric analysis of publications in a specific field and explore the key paths and knowledge inflection points of the evolution by drawing a series of knowledge maps [11–13]. Recently, the co-occurrence analysis and burst detection functions of CiteSpace have received much attention due to the ability to identify topics that have suddenly increased in popularity over time. Thus, it is often used to investigate the research hotspots and emerging trends of a research field based on quantitative analysis [14–17].

In this study, CiteSpace was used to visualize the eligible publications relating prognosis of CHD after PCI from the Web of Science Core Collection (WoSCC) database. Methods of co-occurrence analysis, cluster analysis, timeline analysis, burst term detection and citation analysis were adopted to analyze literature concerning prognosis of CHD after PCI over the period 2004–2022. Knowledge maps was drawn and the research hotspots and emerging trends in this field were extracted and summarized. In short, we explored the knowledge domain, quantitative research mode and development trends regarding prognosis of CHD after PCI to help researchers acquire accurate and complete information about prognosis of CHD after PCI.

## Materials and methods

### Data collection

Data used for bibliometric analysis was collected from the WoSCC database of Thomson Reuters including SCI-Expanded, SSCI, A&HCl, CPCI-S, CPCI-SSH, ESCI, CCR-Expanded and IC. The topic search consisted of index words about prognosis of CHD after PCI was as follows: “prognos*”, “cardiovascular adverse event”, “MACE”, “nomogram”, “predict*”, “percutaneous coronary intervention”, “percutaneous coronary revascularization” and “PCI”. This search produced 14,699 records, including 14,212 articles and reviews about the “cardiac cardiovascular systems”. Literature search time was limited to the period of 2004 to 2022 and eligible publications were downloaded on December 31, 2022. Search records were then exported and transferred to CiteSpace software for further analysis [11]. Each document record included title, abstract, keyword, author, descriptor, citation, identifier, publishing year and publishing organization. Research data involved in this study was obtained from an open-access public database and no ethical issue was involved.

### Inclusion criteria

Inclusion criteria were: (1) Peer-reviewed and published original articles concerning the prognosis of CHD after PCI, including basic and clinical research papers; (2) Reviews on prognosis of CHD after PCI; (3) Articles published between 2004 and 2022; and (4) Articles retrieved from WoSCC.

### Exclusion criteria

Exclusion criteria were: (1) Non-officially published papers; (2) Conference abstracts and proceedings, corrigendum documents; and (3) Repeated publications.

### Quality assessment

Eligible records that met the selection criteria were included in the analysis. By going through the full text of the included materials, unqualified articles were removed while qualified ones were identified. Literature screening was conducted independently by two researchers and discrepancies were resolved by discussion. Otherwise, a third researcher would be involved.

### Visualization analysis

CiteSpace V5.8.R2 (Version 5.8.R2) was used for visualization analysis in this study. In the analysis of co-occurrence or cooperation networks, we typically selected the top 50 articles from each time slice. The time slice parameter was set as 1 year, and the time period was 2004–2022. Pruning methods such as pathfinder, pruning sliced networks and pruning the merged network were used to simplify the network atlas. The included publications were analyzed in terms of authors, institutions and countries. The co-occurrence analysis, cluster analysis, timeline analysis and burst term detection were conducted with keywords as their nodes. The thickness of the line between nodes was positively correlated with the degree of association between nodes. Betweenness centrality (BC) was defined by Freeman in 1977. The BC parameter was calculated by the following Eq. (1) [18].

1 $$BC_{i} = \sum\nolimits_{{i \ne s \ne t}} {\frac{{n_{{st}}^{i} }}{{{\text{g}}_{{st}} }}}$$

In Eq. (1), ${\mathrm{g}}_{st}$ represents the number of shortest paths between node *s* and node *t* and ${\mathcal{n}}_{st}^{i}$ is the number of those paths that pass-through node *i*. The importance of each node in the network can be partially evaluated by the indicator of BC. A node with a high BC (≥ 0.1) was defined as a turning point and marked with a purple circle [12], and the thickness was positively correlated with the BC. The clarity of keyword clustering results was judged by module value (Q value) and average contour value (S value). Clusters with Q value higher than 0.3 were considered to be clear and significant. Clusters with S value higher than 0.5 and 0.7 were considered to be reasonable and have high efficiency and credibility, respectively [13].

## Results

### Publication years and journals

**Fig. 1:**
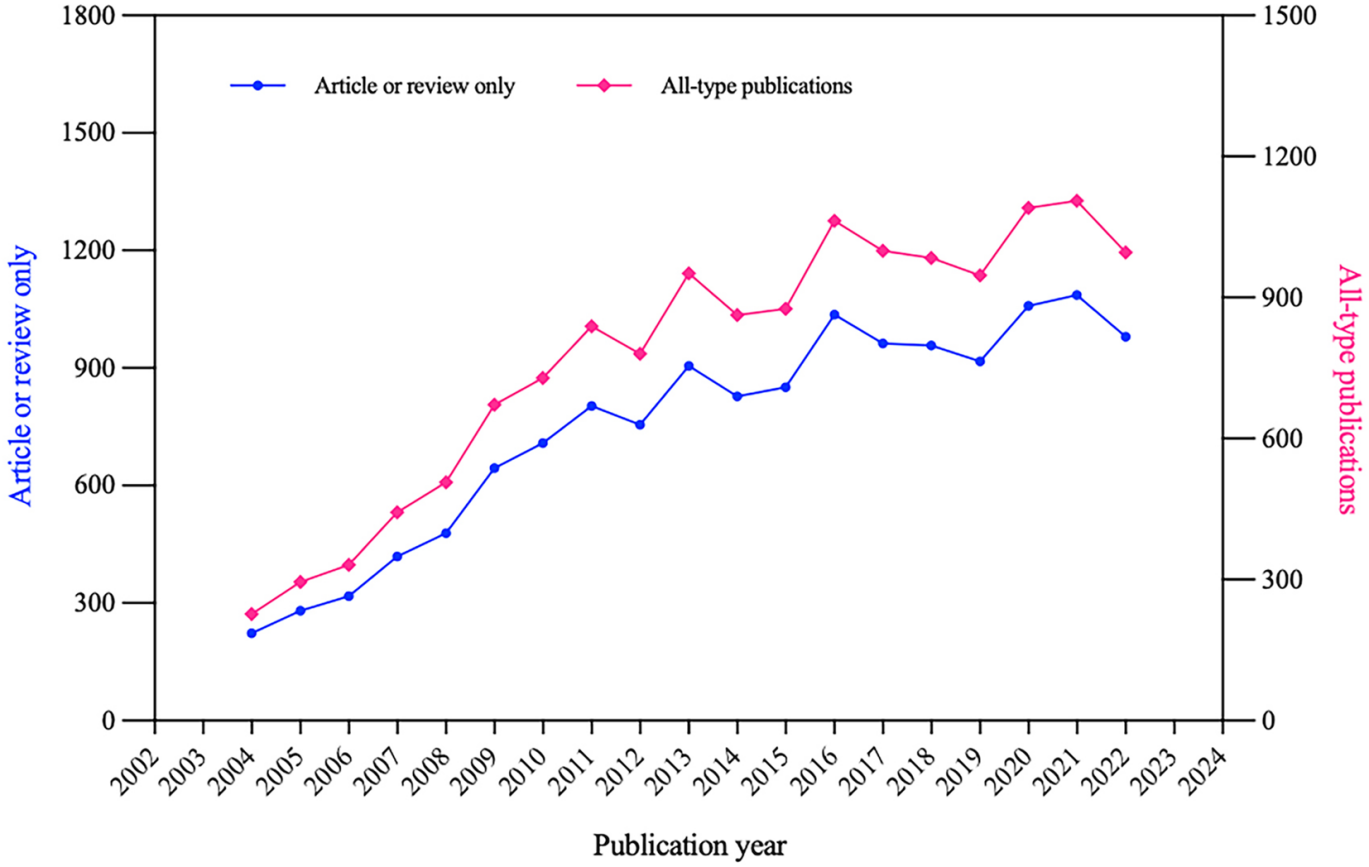
Time sequence of publications regarding prognosis of CHD after PCI between 2004 and 2022 in WoSCC

A total of 14,212 literature that met our selection criteria were obtained by searching the WoSCC database. With a growing number of researches concerning prognosis of CHD after PCI, annual publication production had increased steadily between 2004 and 2022. The number of all-type publications had surged from 227 in 2004 to 996 in 2022 while that of published articles and reviews had increased from 223 to 980, as shown in Fig. 1.

All the articles and reviews relating prognosis of CHD after PCI were published in 153 journals. *The American Journal of Cardiology* ranked first in the number of publications (n = 1053) and it was followed by *Catheterization and Cardiovascular Interventions* (n = 878) and the *International Journal of Cardiology* (n = 682). The top 15 journals with the largest number of publications concerning prognosis of CHD after PCI were listed in Table 1, which could provide references and ideas for investigators to do further research regarding this field.

**Table 1:** Top 15 most productive journals

Journals	Impact factor	The number of published papers
*American Journal of Cardiology*	2.778	1 053
*Catheterization and Cardiovascular Interventions*	2.692	878
*International Journal of Cardiology*	4.164	682
*JACC-Cardiovascular Interventions*	11.196	448
*American Heart Journal*	4.749	410
*Journal of the American College of Cardiology*	24.093	385
*EuroIntervention*	6.534	337
*Coronary Artery Disease*	1.439	334
*Circulation Journal*	2.993	310
*Journal of Invasive Cardiology*	2.022	274
*Circulation-Cardiovascular Interventions*	6.546	270
*Journal of Interventional Cardiology*	2.279	263
*European Heart Journal*	29.983	243
*Journal of the American Heart Association*	5.501	216
*Circulation*	29.690	214

### Co-authorship

**Fig. 2:**
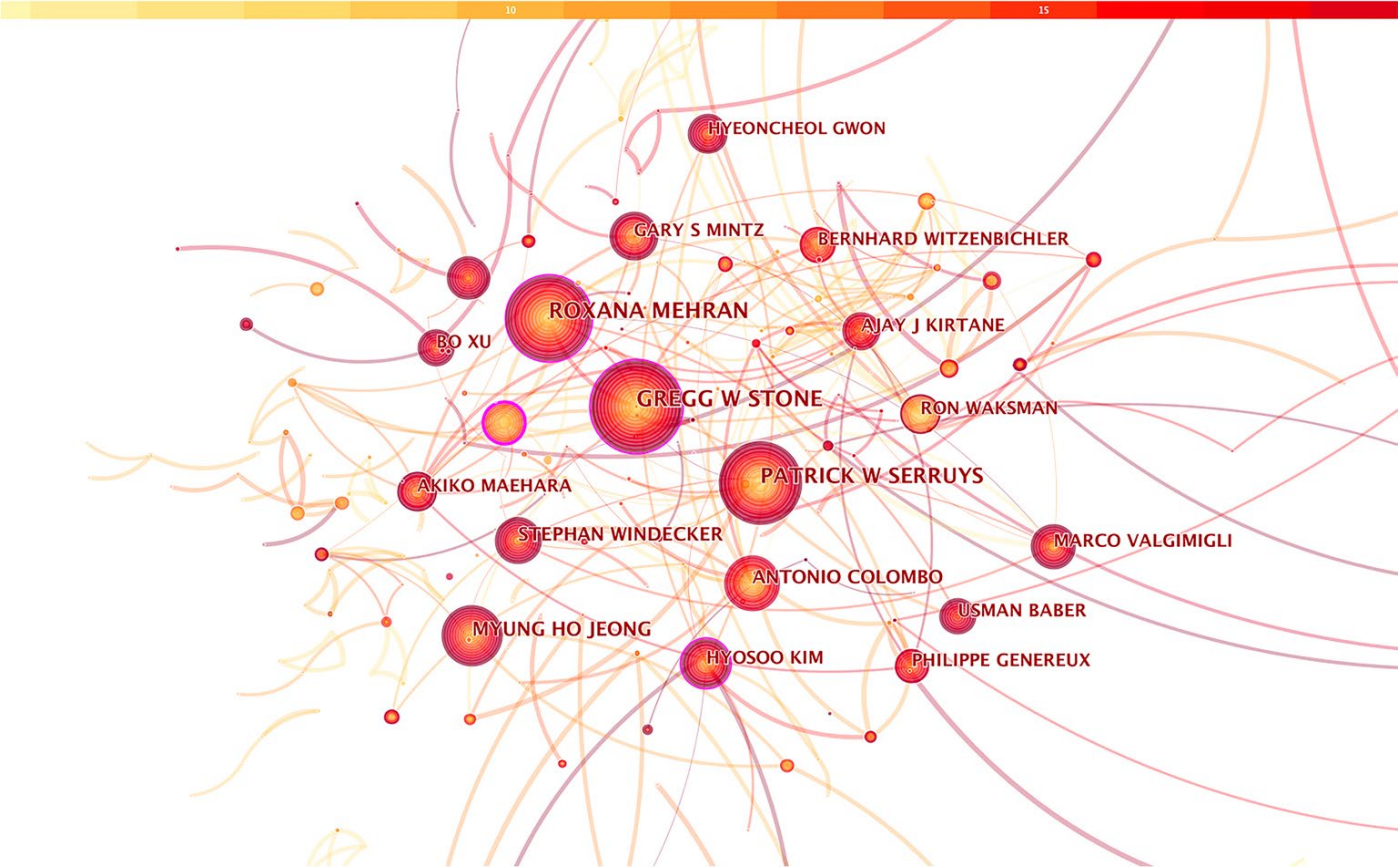
The cooperation network of productive authors

Papers published over the period 2004–2022 were chosen and analyzed as the time slice parameter setting as 1 year and the selection criteria were the top 50 per slice. The co-authorship network is displayed in Fig. 2. Nodes represented authors and the size of each node corresponded the number of publications. Different colored circles represented articles published in different years: yellow represented the earlier publications while red represented recent ones. The shorter the distance between two circles, the more connected between the two authors.

In a result, a total of 1182 unique nodes and 2880 links were generated with a density of 0.0041. Figure 2 demonstrats that the most representative author was Gregg W Stone from the CardioVascular Research Foundation (CVRF) with 368 publications relating prognosis of CHD after PCI, followed by Roxana Mehran from the CVRF with 324 publications and Patrick W Serruys from the National University of Ireland Galway with 246 publications. It could be noticed that many authors tended to work in a relatively stable group as several major clusters of authors were generated and each of these clusters usually had two or more core authors. This analysis could offer personalized scientific research information relating prognosis of CHD after PCI for other investigators. The top 10 authors with the largest number of publications or listed in Table 2. All of them have published more than 100 relevant articles in this field. Among them, the BC of nodes representing three authors, including Gregg W Stone, Roxana Mehran and Hyosoo Kim, was greater than 0.1, suggesting that these three authors had closer contact and communication with other authors in the field of prognosis of CHD after PCI.

**Table 2:** Top 10 most productive authors

Author	Betweenness centrality	The number of published papers
Gregg W Stone	0.11	368
Roxana Mehran	0.15	324
Patrick W Serruys	0.04	246
Myung Ho Jeong	0.02	152
Antonio Colombo	0.07	146
Stephan Windecker	0.06	129
Marco Valgimigli	0.07	108
Gary S Mintz	0.05	105
Hyosoo Kim	0.12	105
Ron Waksman	0.05	102

### Co-institute

**Fig. 3:**
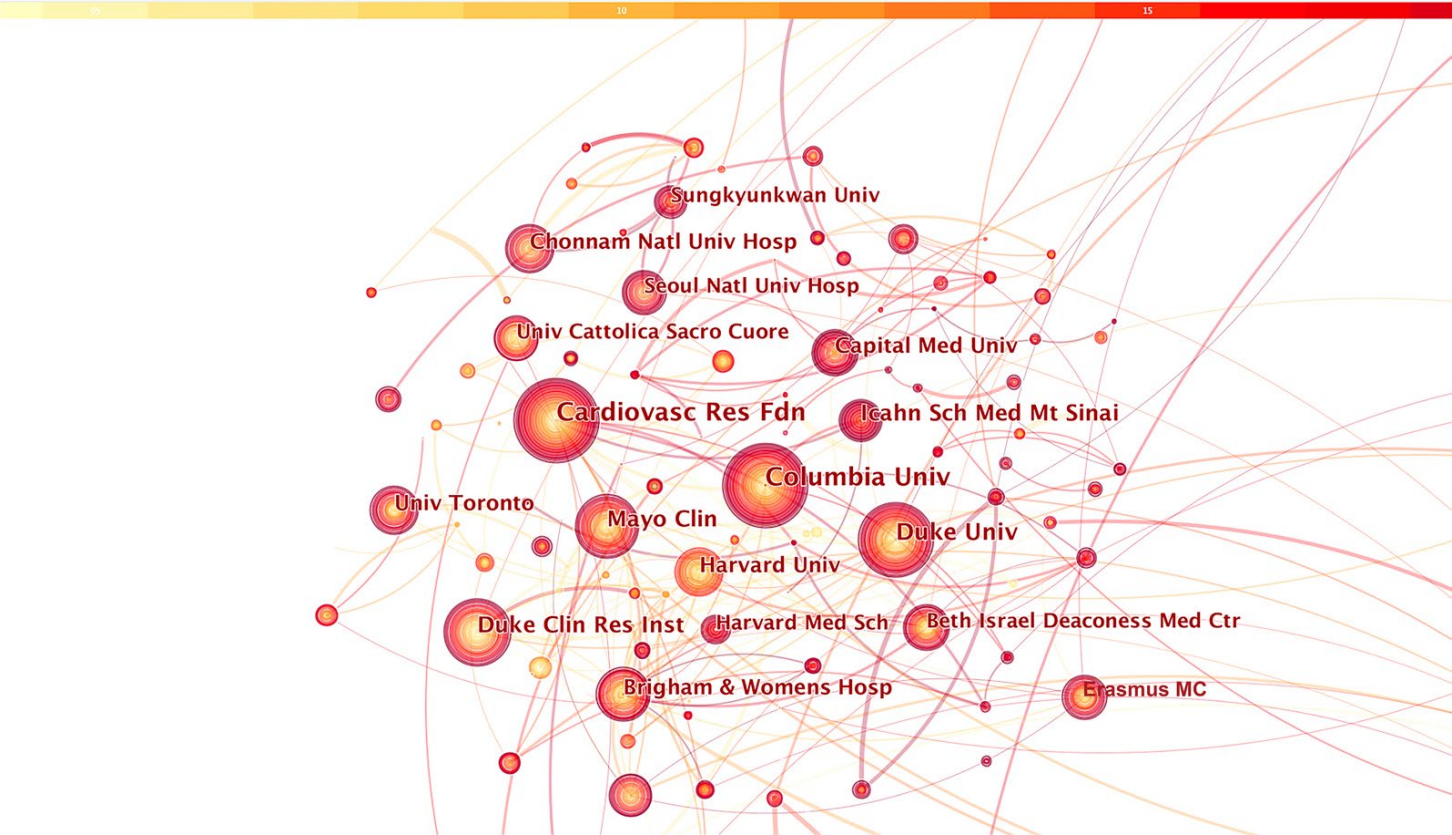
The cooperation network of productive institutes

A cooperation would be considered if two authors’ institutes appear in a same article. CiteSpace software mainly judges cooperation relying on the co-occurrence frequency matrix. Publications over the period 2004–2022 were chosen and analyzed as the time slice parameter setting as 1 year and the top 50 most-cited or -occurring items were chosen from each slice. Figure 3 exhibits co-institutes in the field of prognosis of CHD after PCI. Nodes represented institutes and the size of circles represented the number of papers published by institutes. The shorter the distance between two circles, the greater the cooperation between the two institutes. Purple rings indicated greater BC (no less than 0.1).

In addition, a total of 796 institutes have contributed to these publications relating prognosis of CHD after PCI and 2601 links were generated in the co-institute analysis. As shown in Fig. 3, the Columbia University in the USA was the most representative institute with the largest number of published articles (n = 516), followed by the CVRF in the USA (n = 434), Duke University in the USA (n = 301), Mayo Clinic in the USA (n = 284), Erasmus Medical Center in Rotterdam (n = 243), Icahn School of Medicine at Mount Sinai in the USA (n = 229), Duke Clinical Research Institute in the USA (n = 226), Capital Medical University in China (n = 188), Harvard University in the USA (n = 179) and Brigham and Women’s Hospital in the USA (n = 167). The top 10 institutes with the largest number of publications regarding prognosis of CHD after PCI were listed in Table 3, the BC of all nodes was less than 0.1, indicating insufficient communication and cooperation between these institutes.

**Table 3:** Top 10 most productive institutes

Institute	Betweenness centrality	The number of published papers
Columbia University	0.01	516
CardioVascular Research Foundation	0.05	434
Duke University	0.06	301
Mayo Clinic	0.09	284
Erasmus Medical Center	0.07	243
Icahn School of Medicine at Mount Sinai	0.03	229
Duke Clinical Research Institute	0.04	226
Capital Medical University	0.03	188
Harvard University	0.03	179
Brigham and Women’s Hospital	0.05	167

### Co-country

**Fig. 4:**
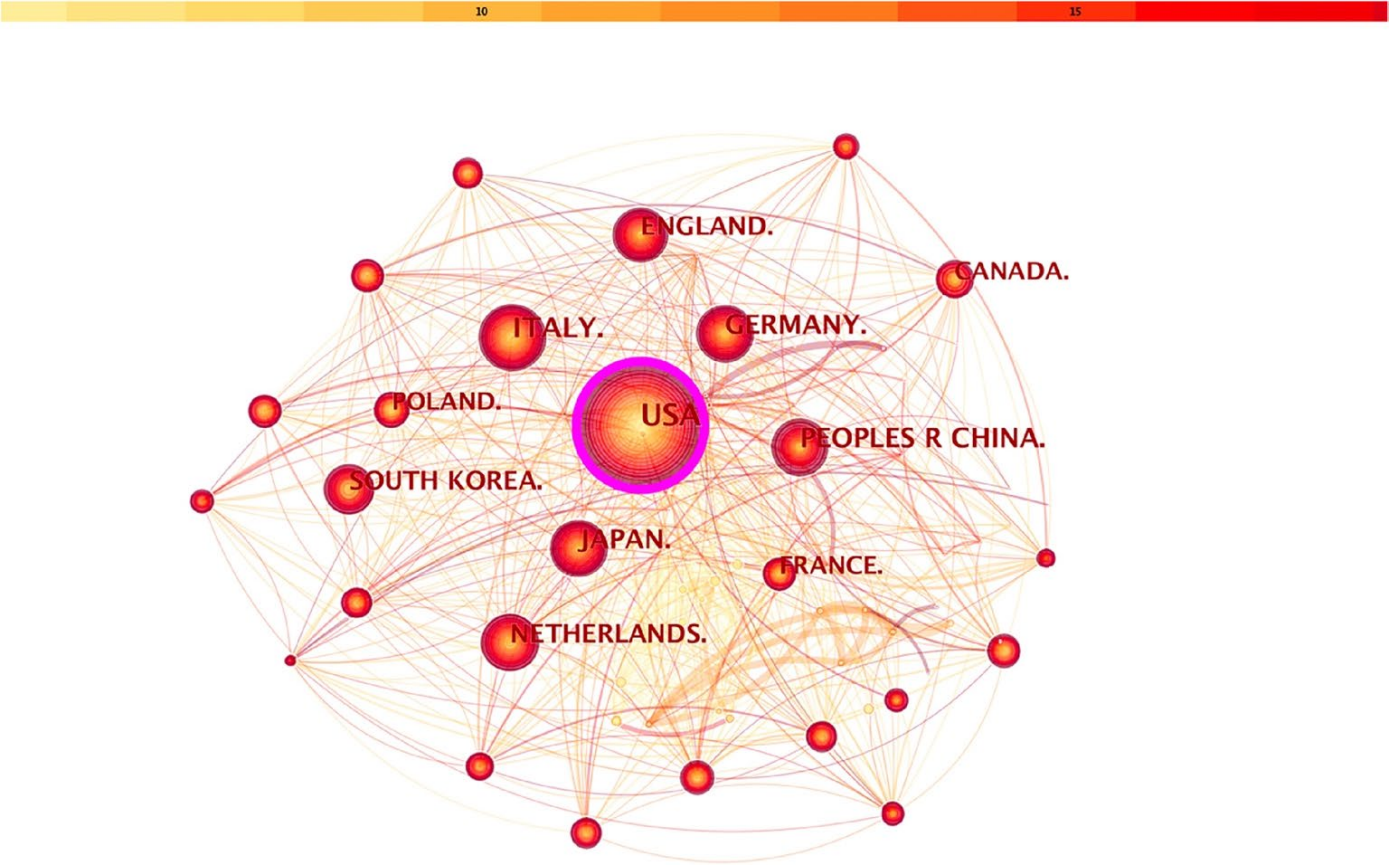
The cooperation network of productive countries

Publications over the period 2004–2022 were chosen and analyzed as the time slice parameter setting as 1 year and the top 50 most-cited or -occurring items were chosen from each slice. Figure 4 displays co-country analysis results concerning prognosis of CHD after PCI. The size of circles represented the number of papers published by countries. The shorter the distance between two circles, the greater the cooperation between the two countries.

Our result showed that a total of 147 countries have contributed to these publications relating prognosis of CHD after PCI. The global productivity ranking was led by the USA with the largest number of published papers (n = 3326), followed by Italy (n = 1355), Japan (n = 1080), China (n = 1075), Germany (n = 937), England (n = 871), Netherlands (n = 856) and South Korea (n = 668). Among them, the top 3 BC ranking was: the USA (0.78), Italy (0.06) and England (0.06). The USA had a closer relationship and cooperation with other countries.

### Co-occurring keywords analysis

**Fig. 5:**
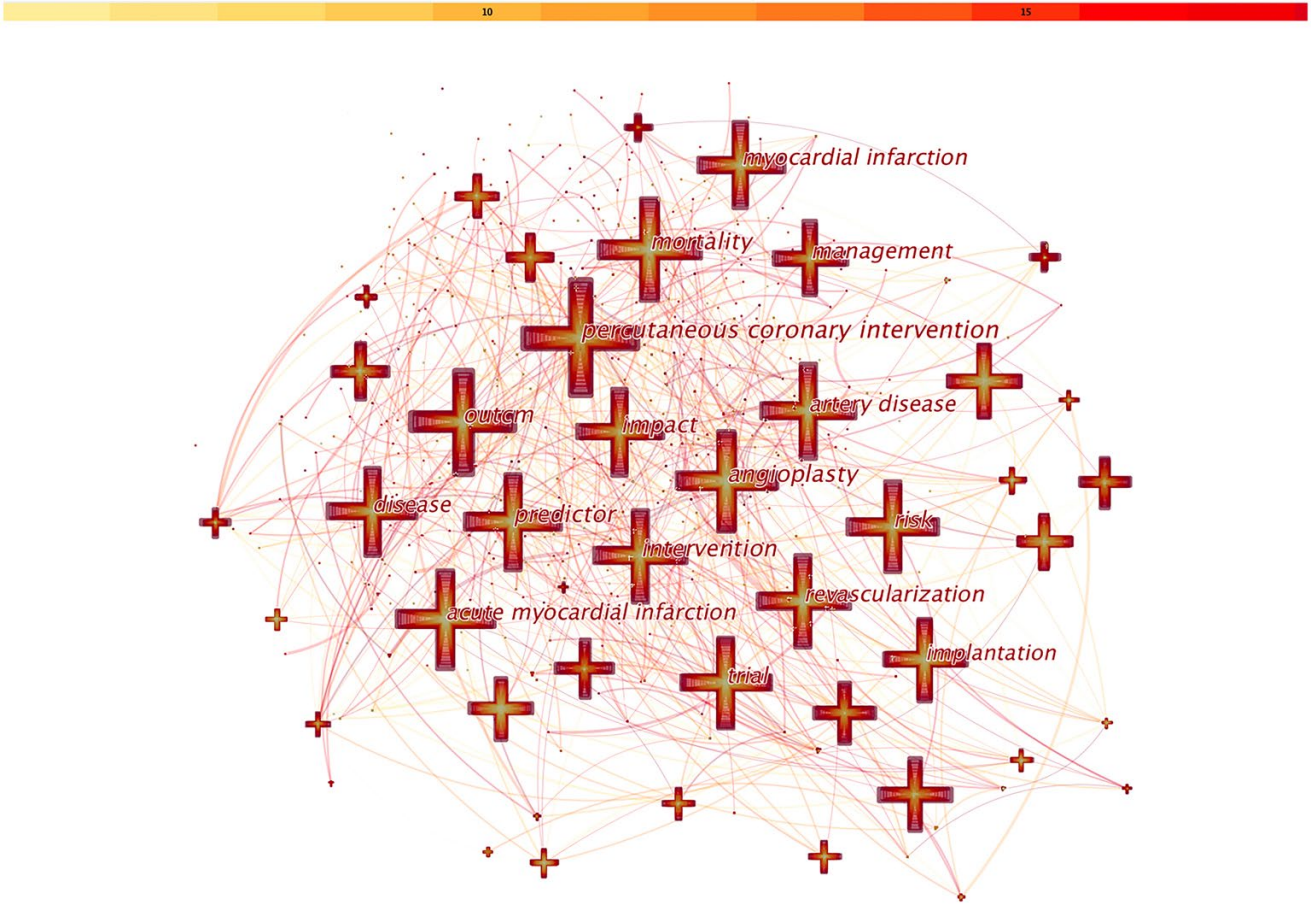
Analysis of co-occurring keywords regarding prognosis of CHD after PCI research

Co-occurring keywords could reflect research hotspots in the field of prognosis of CHD after PCI. Published papers between 2004 and 2022 were chosen and analyzed as the time slice parameter setting as 1 year. As shown in Fig. 5, a simplified co-occurring keyword network was obtained with the pathfinder algorithm. A node represented a keyword and the size of each node was consistent with their co-occurring frequency. The color of co-occurring links between keywords represented the chronological order: oldest in gray and newest in green.

As a result, a total of 932 keywords in 14,212 publications with a net density of 0.0129 were generated. Of the commonly used words, “percutaneous coronary intervention” constituted the highest frequency (n = 2271), followed by “outcome” (n = 1756), “mortality” (n = 1730) and “impact” (n = 1334). Other commonly used words were “predictor” (n = 1324), “intervention” (n = 1310), “angioplasty” (n = 1299), “risk” (n = 1144), “acute myocardial infarction” (n = 1136), “artery disease” (n = 1098). A few of nodes were marked with purple circles, which indicated that they had a high BC. In other words, corresponding keywords represented emerging trends in the field of prognosis of CHD after PCI.

### Timeline analysis

**Fig. 6:**
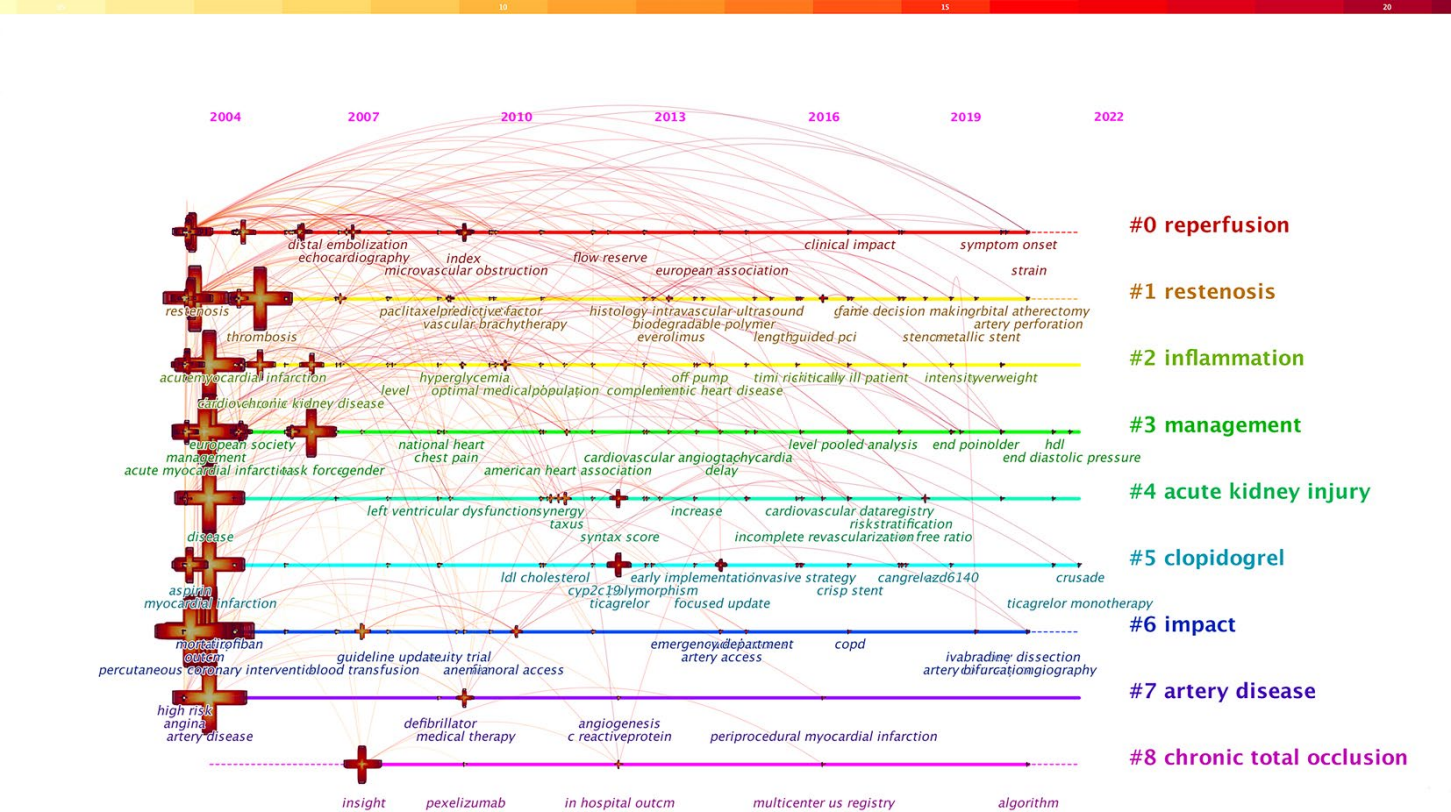
Timeline analysis regarding prognosis of CHD after PCI

Figure 5 was sorted by time zone to obtain Fig. 6 with the year of keyword occurrence as the X-axis and the cluster label as the Y-axis. Figure 6 shows the historical process concerning prognosis of CHD after PCI. Different nodes represented different keywords. The size of nodes was positively correlated with the frequency of keywords. Different colors of nodes represented different times and the colors from cold to warm represented the ages from far to near. It demonstrated that the keywords frequently appeared between 2004 and 2010 were “angioplasty”, “unstable angina”, “restenosis”, “balloon”, “thrombolytic therapy”, “reperfusion” and “bare stent”. After 2010, research hotspots were focused on the exploration of “management”, “cardiac surgical score”, “optical coherence tomography”, “dual antiplatelet therapy”, “ticagrelor”, “nursing analysis”, “drug-eluting stent”, etc.

### Burst term detection

**Fig. 7:**
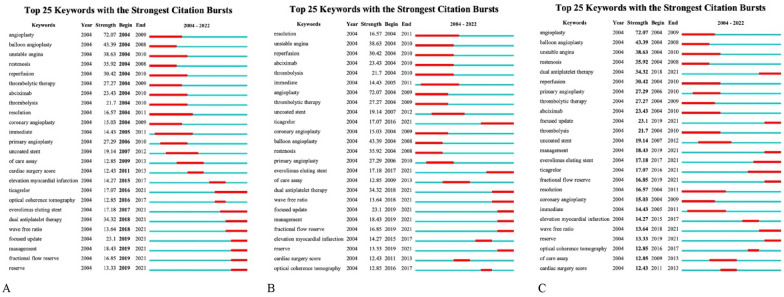
Burst term detection regarding prognosis of CHD after PCI. **A.** Sort by burst onset time **B.** Sort by burst duration** C**. Sort by burst intensity

Burst term detection could detect the research hotspots and potential emerging trends in different periods. “Begin” and “end” indicated the start time and end time of the burst, respectively. “Strength” represented the intensity of the keyword burst and the higher the intensity, the greater the influence [11, 12]. According to the characteristics of eligible publications, the γ parameter was set to 1 and the minimum duration was 2. Finally, a total of 25 burst keywords were obtained, as shown in Fig. 7. In the beginning, more attention was paid to “angioplasty”, “balloon”, “unstable angina”, “restenosis”, “reperfusion”, “thrombolytic therapy”, “abciximab”, etc. Gradually, the focus shifted to “decision making”, “bare stent”, “dual antiplatelet therapy”, “ticagrelor”, “cardiac surgical score”, “optical coherence tomography”, etc. Among them, keywords with longer burst duration were “decision making”, “unstable angina”, “reperfusion”, “angioplasty”, “thrombolytic therapy” and “ticagrelor”. In addition, the burst intensity of “angioplasty”, “balloon”, “unstable angina”, “restenosis”, “dual antiplatelet therapy”, “reperfusion” and “thrombolytic therapy” was higher than others.

### Document co-citation analysis

**Fig. 8:**
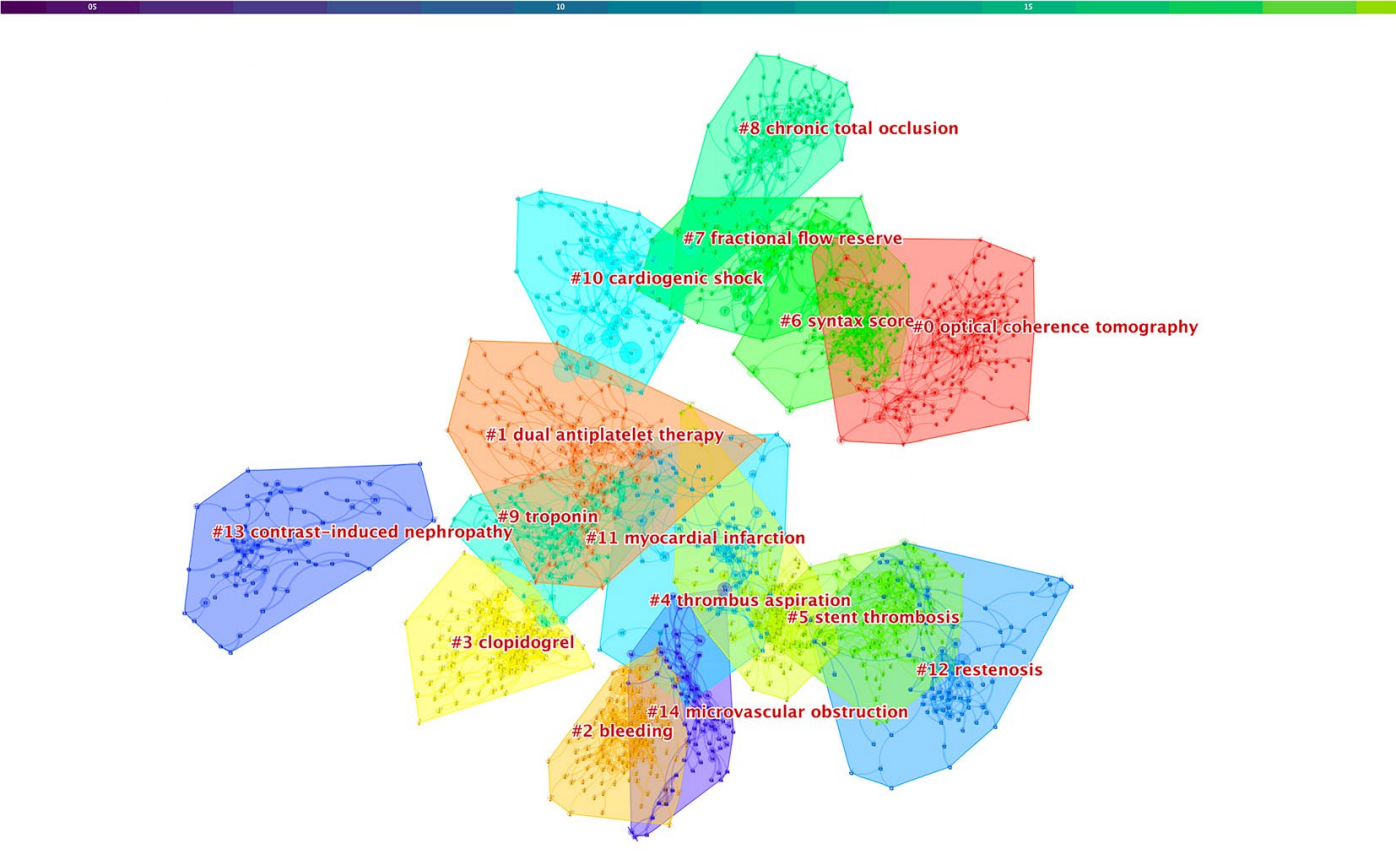
Cluster keywords analysis regarding prognosis of CHD after PCI

Research patterns and emerging trends in the knowledge system in terms of key clusters of cited references were explored. In result, a total of 932 keywords in 14,212 publications were analyzed and visualized by using CiteSpace software. Publications over the period 2004–2022 were chosen and analyzed as the time slice parameter setting as 1 year and the top 50 most-cited or -occurring items were chosen from each slice. The cluster keywords network pruned by pathfinder was generated. Nodes and links represented cited references and co-citation relationships, respectively. The color and thickness of circle indicated the frequency of every node at different periods. Line colors corresponded directly to the time slice: the cold colors represented earlier years while warm ones represented the near years. For example, purple lines represented keywords that appeared in 2004 and green lines visualized recent ones. A cluster cited references analysis network map was shown in Fig. 8.

Figure 8 shows that 15 high connected clusters with Q value of 0.831 and S value of 0.9388, including 1752 unique nodes and 3,656 lines, were generated. These clusters were labeled by indexed terms from their own keywords. To characterize the nature of a cluster, noun phrases were extracted from the keywords in the article that cited the cluster by CiteSpace based on two specialized algorithms—the log-likelihood ratio (LLR) and the latent semantic indexing (LSI). Of the two, LLR generally gave the best results in terms of the uniqueness and coverage of cluster-related topics. The detailed information of the 15 high connected clusters is summarized in Table 4. Accordingly, “optical coherence tomography” was the largest cluster (#0) consisting of 141 members, followed by “dual antiplatelet therapy” (#1) with 120 members and “bleeding” (#2) with 113 members.

**Table 4:** The largest 15 clusters of keywords regarding prognosis of CHD after PCI

Cluster ID	Size	Mean (cite year)	LLR	LSI
#0	141	2013	Optical coherence tomography	Percutaneous coronary intervention
#1	120	2015	Dual antiplatelet therapy	Optical coherence tomography
#2	113	2009	Bleeding	Coronary artery disease
#3	109	2007	Clopidogrel	Acute coronary syndrome
#4	109	2003	Thrombus aspiration	Myocardial infarction
#5	107	2007	Stent thrombosis	ST-segment elevation
#6	101	2012	SYNTAX score	Major coronary arteries
#7	97	2014	Fractional flow reserve	Fractional flow reserve
#8	97	2012	Chronic total occlusion	Chronic total occlusion
#9	95	2003	Troponin	Ventricular hypertrophy
#10	84	2013	Cardiogenic shock	Multivessel coronary artery disease
#11	83	2005	Myocardial infarction	Platelet aggregation inhibitors
#12	82	2001	Restenosis	Coronary stent
#13	73	2002	Contrast-induced nephropathy	Risk assessment
#14	73	2011	Microvascular obstruction	Antiplatelet drug

*LLR* log-likelihood ratio, *LSI* latent semantic indexing

### Emerging trends

A significant increase in research interests concerning prognosis of CHD after PCI were highlighted by publications with citation bursts. Table 5 shows the top 15 references with the strongest citation values over the period 2004–2022, including 8 guidelines and expert consensus (3 guidelines for the management of acute coronary syndromes [19–21], 1 guideline for PCI [22], 1 guideline on myocardial revascularization [23], 1 consensus report for clinical end points in coronary stent trials [24], 1 consensus report for bleeding definitions for cardiovascular clinical trials, [25] 1 consensus report for the definition of myocardial infarction [26]), 6 clinical trials (3 randomized controlled trials on the efficacy of anti-coagulant/anti-platelet drugs in acute coronary syndromes [27–29], 2 multi-center randomized controlled trial of the efficacy and safety of stent implantation [30, 31], 1 randomized controlled trial comparing the efficacy and safety of PCI and coronary artery bypass grafting (CABG) in severe coronary artery disease (CAD) [32]) and a systematic review comparing the efficacy of angioplasty and thrombolytic therapy on acute myocardial infarction [33]. Among the 15 articles mentioned above, four articles were from cluster #10 and three articles were from cluster #6. Besides, there are two articles in each of clusters #2, #3 and #5, and one article in each of clusters #11 and #12. Four of the top five articles were published before 2010.

**Table 5:** Top 15 references with high citation values regarding prognosis of CHD after PCI

Citation counts	Author	Year	Betweenness centrality	Source	Cluster ID
631	Cutlip DE	2007	0.01	CIRCULATION, 115, 2344	#5
531	Steg PG	2012	0.02	EUR HEART J, 33, 2569	#10
528	Windecker S	2014	0.01	EUR HEART J, 35, 2541	#6
428	Ibanez B	2018	0.07	KARDIOL POL, 76, 229	#10
390	Thygesen K	2012	0.03	J AM COLL CARDIOL, 60, 1581	#10
316	Serruys PW	2009	0.03	NEW ENGL J MED, 360, 961	#6
299	Levine GN	2011	0.02	J AM COLL CARDIOL, 58	#6
293	Mehran R	2011	0.02	CIRCULATION, 123, 2736	#2
280	Roffi M	2016	0.03	EUR HEART J, 37, 267	#10
254	Iakovou I	2005	0.04	JAMA—J AM MED ASSOC, 293, 2126	#5
253	Moses JW	2003	0.04	NEW ENGL J MED, 349, 1315	#12
235	Keeley EC	2003	0.03	LANCET, 361, 13	#11
218	Stone GW	2008	0.03	NEW ENGL J MED, 358, 2218	#2
212	Wiviott SD	2007	0.01	NEW ENGL J MED, 357, 2001	#3
209	Wallentin L	2009	0.03	NEW ENGL J MED, 361, 1045	#3

Table 6 shows the top 15 references with the strongest burst values between 2004 and 2022, including 3 guidelines for the management of acute coronary syndromes [19–21], 2 guidelines on myocardial revascularization [23, 34], 2 consensus reports for the definition of myocardial infarction [26, 35], 1 guideline for PCI [22], 1 consensus report for clinical end points in coronary stent trials [24], 1 consensus report for bleeding definitions for cardiovascular clinical trials [25], 4 multi-center randomized controlled trials of the efficacy and safety of stent implantation, [30, 31, 36, 37] and a systematic review comparing the efficacy of angioplasty and thrombolytic therapy on acute myocardial infarction [33]. Among them, four articles were from cluster #1 and three articles were from cluster # 12. In addition, there are two articles in each of clusters #5 and #6, and one article in each of clusters #2, #7, #11 and #17. As we can see, the details in Table 6 were largely similar to the information in Table 5, indicating these publications stood for the research hotspots and trends with regard to prognosis of CHD after PCI and played a vital role in guiding future research directions in this field.

**Table 6:** Top 15 references with high burst values regarding prognosis of CHD after PCI

Burst	Author	Year	Betweenness centrality	Source	Cluster ID
186.89	Ibanez B	2018	0.07	KARDIOL POL, 76, 229	#10
147.93	Windecker S	2014	0.01	EUR HEART J, 35, 2541	#6
144.31	Cutlip DE	2007	0.01	CIRCULATION, 115, 2344	#5
125.98	Steg PG	2012	0.02	EUR HEART J, 33, 2569	#10
99.99	Neumann FJ	2019	0.00	EUR HEART J, 40, 87	#7
91.52	Moses JW	2003	0.04	NEW ENGL J MED, 349, 1315	#12
84.96	Keeley EC	2003	0.03	LANCET, 361, 13	#11
84.54	Thygesen K	2012	0.03	J AM COLL CARDIOL, 60, 1581	#10
83.65	Roffi M	2016	0.03	EUR HEART J, 37, 267	#10
79.1	Morice M	2002	0.01	NEW ENGL J MED, 346, 1773	#12
70.08	Mehran R	2011	0.00	CIRCULATION, 123, 2736	#2
68.47	Iakovou I	2005	0.02	JAMA—J AM MED ASSOC, 293, 2126	#5
66.35	Thygesen K	2018	0.04	GLOB HEART, 13, 305	#17
62.06	Stone GW	2004	0.04	NEW ENGL J MED, 350, 221	#12
60.33	Levine GN	2011	0.02	J AM COLL CARDIOL, 58	#6

## Discussion

### Co-occurrence analysis

Based on the research methods of bibliometric studies and characteristics of cardiology, our study explored the scientometric characteristics regarding prognosis of CHD after PCI by analyzing and visualizing relevant literature published over the period 2004–2022. In this study, a total of 14,212 eligible publications concerning prognosis of CHD after PCI were extracted and analyzed from the WoSCC database. Co-occurrence analysis, cluster analysis, timeline analysis, burst term detection and citation analysis were conducted to investigate the knowledge domain and development trends in the field of prognosis of CHD after PCI by using CiteSpace V5.8.R2 (Version 5.8.R2).

Our findings demonstrated that annual publication production regarding prognosis of CHD after PCI had increased steadily between 2004 and 2022 and the number of published articles was linearly correlated with the published time. In 2022, the number of publications in this field reached 1079 and they were published in 153 different journals under the name of 1182 different authors. Besides, a total of 796 institutes and 147 countries have contributed to these publications. According to the number of publications, “American Journal of Cardiology”, “Catheterization and Cardiovascular Interventions”, “International Journal of Cardiology”, “JACC-Cardiovascular Interventions”, “American Heart Journal”, “Journal of the American College of Cardiology”, “EuroIntervention”, “Coronary Artery Disease”, “Circulation Journal”, “Journal of Invasive Cardiology” were journals of academic importance. In addition, the global productivity ranking was led by the USA with 3326 publications.

The co-authorship network map showed that 1182 researchers contributed to this field and closely connected and cooperated with each other. Among them, the BC of nodes representing the three authors, including Gregg W Stone, Roxana Mehran and Hyosoo Kim was greater than 0.1. Each of them published more than 100 articles, suggesting that these three authors have had a significant impact in the field of prognosis of CHD after PCI and had closer cooperation with other authors. Moreover, Professor Gregg W Ston’s team mainly focused on exploring the efficacy and safety of revascularization and the characteristics of susceptible population. Their study demonstrated that large periprocedural infarctions (signified by new Q waves and CK-MB) were an important risk factor for poor prognosis after PCI [38]. Besides, compared to angioplasty, the authors found that PCI could lower the post-procedure recurrence rate of cardiovascular adverse events and it could be used as a routine reperfusion strategy for acute myocardial infarction [39]. Moreover, they also found that anti-coagulation with bivalirudin alone after PCI, as compared to heparin plus glycoprotein IIb/IIIa inhibitors, resulted in significantly-reduced of major bleeding and net adverse clinical events in 4 weeks [28]. Professor Roxana Mehran’s group focused on in-stent restenosis after PCI. It has shown that repeat stenting for the treatment of focal in-stent had a higher rate of post-procedure CK-MB elevation but similar long-term clinical results compared with angioplasty alone [40]. Meanwhile, lesion length remained to be a powerful predictor of recurrent in-stent restenosis [41]. Mehran et al. participated in developing and forming a consensus on the standardized bleeding definitions for cardiovascular clinical trials [25]. Professor Hyosoo Kim’s team paid more attention to the efficacy and safety of different types of stent implantation methods and dual anti-platelet therapy strategies after PCI. Their team confirmed that durable polymer drug-eluting stents were no worse than biodegradable polymer drug-eluting stents in terms of patient-oriented composite outcomes after 12-month follow-up after PCI [42]. It showed that a prasugrel-based de-escalation strategy at 1 month after PCI reduced the risk of net clinical outcomes up to 1 year in patients after PCI [43].

The co-institute network map showed that a total of 796 institutes have contributed to these publications relating prognosis of CHD after PCI and they had a close communication with each other. The top 10 institutes with the largest number of published papers were Columbia University, the CVRF, Duke University, Mayo Clinic, Erasmus Medical Center, Icahn School of Medicine at Mount Sinai, Duke Clinical Research Institute, Capital Medical University, Harvard University, and Brigham and Women’s Hospital. Each of them had published more than 150 relevant articles yet. It was worth noting that eight of the top 10 institutes were from the USA, which fully reflected the international academic leadership of the USA concerning prognosis of CHD after PCI. However, many papers concerning prognosis of coronary heart disease after PCI have been published, the academic cooperation among international institutes still need to be strengthened. Furthermore, the co-country analysis suggested that the USA had the largest number of publications and the highest BC, with a huge purple circle around the node, showing that the USA was the backbone in this field and assumed the role of network hub. Moreover, Italy, Japan, China, Germany, England, Netherlands and South Korea also played an essential role with regard to prognosis of CHD after PCI.

### Knowledge domain and development trends

Keywords could highly summarize and condense the topic of an article [11, 12]. High-frequency keywords in different periods reflected the change of research hotspots in a field [13]. Clustering analysis could detect the structural features among clusters, highlighted key nodes and important connections and revealed a research topic and its evolution process in the knowledge domain [11]. Combined with the top 15 high-frequency keywords, the results of keyword co-occurrence analysis could be further summarized as the following six hot topics: (1) the main research object in this field was CHD patients after PCI (keywords: percutaneous coronary intervention, acute myocardial infarction, arterial disease, myocardial infarction, disease); (2) a series of relevant studies were conducted (keywords: trials); (3) the efficacy and safety of treatment strategies in this field were explored and compared (keywords: angioplasty, revascularization); (4) risk factors were analyzed in susceptible populations (keywords: predictors, risk); (5) to guide clinical decisions (keywords: intervention, management); and (6) to further improve the long-term prognosis of patients (keywords: outcome, mortality, impact). It could be inferred that the distribution of research emphasis was comprehensive and systematic in international publications. And the top 5 high-frequency keywords were “percutaneous coronary intervention”, “outcome”, “mortality”, “impact” and “predictors”, which further emphasized the current research hotspots and frontiers concerning prognosis of CHD after PCI. Between 2004 and 2010, “angioplasty”, “unstable angina”, “restenosis”, “balloon”, “thrombolytic therapy”, “reperfusion” and “bare stent” were the research hotspots. Afterwards, more and more studies focused on the exploring of “management”, “cardiac surgical score”, “optical coherence tomography”, “dual antiplatelet therapy”, “ticagrelor”, “nursing analysis”, “drug-eluting stent” over the past 10 years. Furthermore, the frontiers in prognosis of CHD after PCI mainly involved “decision making”, “reperfusion”, “angioplasty”, “balloon”, “unstable angina”, “dual antiplatelet therapy”, “cardiac surgical score”, “restenosis”, “reperfusion”, “thrombolytic therapy”, etc., indicating that it might be a future research trend to pay more attention to early risk assessment, post-procedure secondary prevention and complications in CHD patients after PCI. Among the cited articles with high citation and high burst values, most publications were categorized into clustering #12 restenosis, #10 cardiogenic shock, #6 SYNTAX score and #5 stent thrombosis, representing the research frontiers in this field. Of the four clusters, #6 was the largest cluster containing 101 members, which mainly focused on the exploring of the impact of CAD severity score regarding prognosis of CHD after PCI, as represented by the SYNTAX score. Clusters #5, #10 and #12 were concerned with adverse cardiovascular events and risk factors after PCI for CHD patients. In brief, it could be seen that a growing number of published papers were centered on the efficacy and safety of PCI therapy and risk factors for poor prognosis after PCI.

SYNTAX score was used to evaluate vessels with a diameter ≥ 1.5 mm using the method of 16-segment coronary artery tree, which could not only describe the anatomical structure of the coronary artery, but also might be useful to guide revascularization in high-risk patients for clinicians. [5] According to the revascularization guideline [5], the SYNTAX score remained the most widely used and validated risk score to guide the choice of revascularization in patients with multi-vessel disease. For patients with mild-complexity CAD (0–22 score), PCI or CABG could be selected on account of individual characteristics and the will of both doctors and patients; for those with moderate-complexity CAD (23–32 score), both clinical characteristics and comorbidities should be taken into consideration while choosing the treatment method; and for those with high-complexity CAD (score ≥ 33), it was reasonable to choose CABG over PCI to confer a survival advantage. In addition, studies have found that CABG was a better choice for patients with severe CAD, such as three-vessel disease or left main artery disease, which resulted in lower rates of the combined endpoint of major adverse cardiac or cerebrovascular events at 1 year [32]. Furthermore, it was confirmed that some clinical and surgery-related factors were important influencing factors of post-procedure thrombosis [31, 44, 45]. Premature discontinuation of anti-platelet drugs, renal failure, coronary bifurcation disease, diabetes and reduced ejection fraction were high-risk factors for thrombosis in patients after PCI [31, 46]. More exactly, non-responsiveness to clopidogrel was a strong independent predictor of stent thrombosis [47], and patients with large-scale CAD also had a high incidence of in-stent restenosis and a worse prognosis after PCI [41]. Three multi-center, large-sample, randomized, double-blind, controlled trials showed that drug-eluting stents were safer than bare stents, with lower rates of stent restenosis and cardiovascular adverse events [30, 36, 37]. In addition, a previous study confirmed that the length of stented segment was independently associated with the incidence of stent thrombosis and death or myocardial infarction after drug-eluting stents implantation and the value of stent length ≥ 31.5 mm was a threshold for the prediction of stent thrombosis [48].

## Limitations

The global research regarding prognosis of CHD after PCI has expanded rapidly over the past 20 years. To our knowledge, this is a first bibliometric study to detect the research hotspots and emerging trends in the field of prognosis of CHD after PCI by using CiteSpace software. However, certain limitations of this study need to be addressed. Firstly, it is difficult to obtain enough and effective information to reveal the rule and predict the trend and hotspots in the field because of the limited number of eligible publications. Secondly, all included publications written in English were retrieved from the WoSCC database, therefore, it might not fully reflect the global trends in this field. Thirdly, bibliometrics software cannot distinguish the real contribution of different authors in a complex cooperative relationship, but could only analyze the number of articles provided by the authors in this field. Thus, relevant software and analytical techniques need to be developed to expand the scope of inclusion and obtain more comprehensive research papers in the future.

## Conclusion

In this study, CiteSpace was used to analyze the distribution characteristics of publication outputs, the co-occurrence of subject categories, the international cooperation of countries and the evolution concerning prognosis of CHD after PCI over the period 2004–2022. Based on the analysis of 14,212 eligible publications from the WoSCC database, the vital characteristics regarding prognosis of CHD after PCI were obtained. It indicated that efficacy and safety of different types of stents, the risk factors of restenosis and thrombotic events after PCI, early risk assessment, and secondary prevention and complications of patients with CHD after PCI were research hotspots and frontier topics in the area, providing a systematic overview of the worldwide prognosis of CHD after PCI research and promoting a better understanding of the knowledge domain and development trends in this field in the past 20 years.

## Data Availability

All data generated or analyzed during this study are included in this published article and its additional information files. Further inquiries can be directed to the corresponding author.
